# Evaluating the bioequivalence and safety of liraglutide injection *versus* Victoza^®^ in healthy Chinese subjects: a randomized, open, two-cycle, self-crossover phase I clinical trial

**DOI:** 10.3389/fphar.2023.1326865

**Published:** 2023-12-22

**Authors:** Chao Liu, Hongrong Xu, Fei Yuan, Hanjing Chen, Lei Sheng, Weili Chen, Haisong Xie, Hongmei Xu, Xuening Li

**Affiliations:** ^1^ Department of Clinical Pharmacology, Zhongshan Hospital, Fudan University, Shanghai, China; ^2^ Hangzhou Zhongmei Huadong Pharmaceutical Co., Ltd., Hangzhou, China

**Keywords:** glucagon-like peptide-1, liraglutide, pharmacokinetics, safety, type 2 diabetes, bioequivalence

## Abstract

**Background:** Liraglutide is an acylated glucagon-like peptide-1 (GLP-1) analog, and its pharmacokinetic and pharmacodynamic properties as a GLP-1 receptor (GLP-1R) agonist make it an important therapeutic option for many patients with type 2 diabetes mellitus. This study compared the bioequivalence and safety of liraglutide with the originator product in healthy Chinese adult subjects.

**Methods:** Subjects (N = 36, both sexes) were randomized in a 1:1 ratio into two groups (18 cases each) for a two-cycle, self-crossover trial. Each cycle involved a single subcutaneous injection of the test and reference drugs, with a washout period of 14 days. The plasma drug concentration was quantified by liquid chromatography-tandem mass spectrometry (LC-MS/MS). The main pharmacokinetic parameters were statistically analyzed to assess drug bioequivalence. Furthermore, the safety of the drugs was assessed throughout the trial.

**Results:** The geometric mean ratios of C_max_, AUC_0-t_, and AUC_0-∞_ were 103.73%, 103.01%, and 103.03%, respectively, and their 90% confidence intervals (CIs) were consistent with the range of 80.00%–125.00%, indicating that the two formulations had similar pharmacokinetics. Meanwhile, safety results showed that both drugs were well tolerated.

**Conclusion:** Studies have shown that the test drug has similar bioequivalence and safety to the reference drug.

**Clinical trial registration:** (http://www.chinadrugtrials.org.cn/index.html), identifier (CTR20171303).

## 1 Introduction

Diabetes mellitus (DM) is one of the largest health burdens in the world (Ong et al., 2023; Qiu et al., 2023), with the global prevalence of diabetes increasing dramatically from 3.2% in 1990 to 6.1% in 2021 and the number of people with diabetes reaching an estimated 529 million and climbing; the prevalence of diabetes is projected to rise dramatically to nearly 10% globally by 2050, with the total number of people with diabetes potentially topping 1.3 billion (Ogurtsova et al., 2022; Ong et al., 2023). Corresponding global health expenditures are $966 billion and are expected to exceed $105.4 billion by 2045 (Sun et al., 2022; Ong et al., 2023). Type 2 DM is the most common form of diabetes, which is often caused by a combination of factors that lead to decreased pancreatic islet function and even insulin resistance, which in turn leads to a series of homeostatic imbalances in the internal environment such as disorders of glucose metabolism, water metabolism, and electrolyte metabolism (Nauck et al., 2021b; Demir et al., 2021; Ahmad et al., 2022). Due to hyperglycemia and metabolic syndrome, patients are prone to microvascular complications, including retinopathy, nephropathy, and neuropathy, and macrovascular complications, including cardiovascular, cerebrovascular, and peripheral vascular diseases (Nauck et al., 2021b; Demir et al., 2021). The current primary treatment strategy is to improve the patient’s pathophysiologic deficits and mitigate cardiac and renal risk while considering the control of blood glucose levels (Nauck et al., 2021b; DeMarsilis et al., 2022).

GLP-1 is an endogenous incretin hormone secreted primarily by intestinal L-cells (Drucker, 2006; Baggio and Drucker, 2007; Müller et al., 2019), which is involved in the regulation of glucose homeostasis through GLP-1R, a G-protein-coupled receptor, in a variety of ways, mainly including stimulation of insulin secretion in a glucose-dependent manner, inhibition of glucagon secretion, reduction of appetite, slowing down of gastric emptying (Drucker and Nauck, 2006; Hare et al., 2010), enhancement of insulin sensitivity (Fernández-Millán et al., 2016; Capuani et al., 2018; Jiang et al., 2018), and reduction of insulin resistance (Jiang et al., 2018). However, endogenous GLP-1 is easily degraded by dipeptidyl dipeptidase-4 (Vahl et al., 2003), making its effective half-life only a few minutes (Drucker, 2003). Liraglutide is an acylated GLP-1 analog, and its pharmacokinetic and pharmacodynamic properties as a GLP-1R agonist make it an important therapeutic option for many patients with type 2 diabetes (Jacobsen et al., 2016; Khera et al., 2016; Tamborlane et al., 2019). Liraglutide was first developed by Novo Nordisk and was approved by the EMA and FDA, respectively, in June 2009 and January 2010 (Knudsen and Lau, 2019; Nauck et al., 2021a) under the trade name Victoza^®^ and can generally be used in combination with first-line non-pharmacological treatment for glycemic control regimens, or in patients whose blood glucose levels cannot be controlled using metformin, or in combination with metformin or sulfonylurea (Meier, 2012; Knudsen and Lau, 2019; Tamborlane et al., 2019; Nauck et al., 2021a; DeMarsilis et al., 2022). Additional clinical studies have shown that liraglutide has significant benefits in delaying β-cell failure, reducing cardiovascular complication control in diabetes, and improving lipid metabolism (Honigberg et al., 2020; Nielsen et al., 2020). Of note, Liraglutide’s weight loss indication was approved for marketing in Europe and the United States in 2014 and 2015, respectively (Bray et al., 2016).

The test drug in this study was provided by Hangzhou Zhongmei Huadong Pharmaceutical Co., Ltd. and Hangzhou Jiuyuan Gene Engineering Co., Ltd. The biosimilar was manufactured using a different production protocol from the original manufacturer, using an *Escherichia coli* expression system to produce the peptide chain structure of liraglutide and then modifying the fatty acid side chain *in vitro*. To date, the trial drug liraglutide injection “Lilupin” has been approved for marketing by the National Medical Products Administration (NMPA) in China in March 2023 for adults with type 2 diabetes. This trial is a comparative study of the pharmacokinetics and safety of liraglutide injection with the original research product Victoza^®^ in healthy subjects in China.

## 2 Methods

### 2.1 Materials

The test drug was liraglutide injection produced by Hangzhou Jiuyuan Gene Engineering Co. (Specification: 3 mL: 18 mg. Batch Number: s20160901), China, and the reference drug liraglutide injection was produced by Novo Nordisk, Denmark (Specification: 3 mL: 18 mg. Batch Number: GP51912).

### 2.2 Subjects

The study subjects were healthy Chinese male and female populations aged 18–45 years, weighing ≥50 kg with a body mass index of 19–26 kg/m^2^. After being fully informed and signing a written informed consent form, the subjects voluntarily underwent a series of examinations, including demographic information, medical history, surgical history, allergy history, tobacco and alcohol history, medication history, physical examination, height, weight, vital signs, electrocardiogram, laboratory tests (routine blood, urine, blood biochemistry, coagulation, thyroid function, blood pregnancy in women, and infectious disease screening), urine drug screening, and alcohol breath test. Inclusion criteria included no history of significant organ disease, physical examination, vital signs, laboratory and all related tests were normal or abnormal without clinical significance, and those who were considered eligible in the judgment of the clinical research physician. All subjects were enrolled only if they met the inclusion criteria and did not meet the exclusion criteria. Details of the exclusion criteria are provided in the Supplementary Material.

### 2.3 Study design and ethics

This study was completed in the Phase I Clinical Trial Ward of Zhongshan Hospital, Fudan University. The trial is registered in the Drug Clinical Trial Registration and Information Publication Platform (http://www.chinadrugtrials.org.cn/index.html # CTR20171303) of the China Drug Administration. The trial passed the ethical review by the Medical Ethics Committee of Zhongshan Hospital, Fudan University (Approval No.: 2017-093). The trial process strictly adhered to the ethical guidelines for human medical research of the Declaration of Helsinki, ICH/GCP, the Code of Practice for the Quality Management of Pharmaceutical Clinical Trials, the Measures for Ethical Review of Biomedical Research Involving Human Beings, and the corresponding requirements of domestic laws and regulations.

**FIGURE 1:**
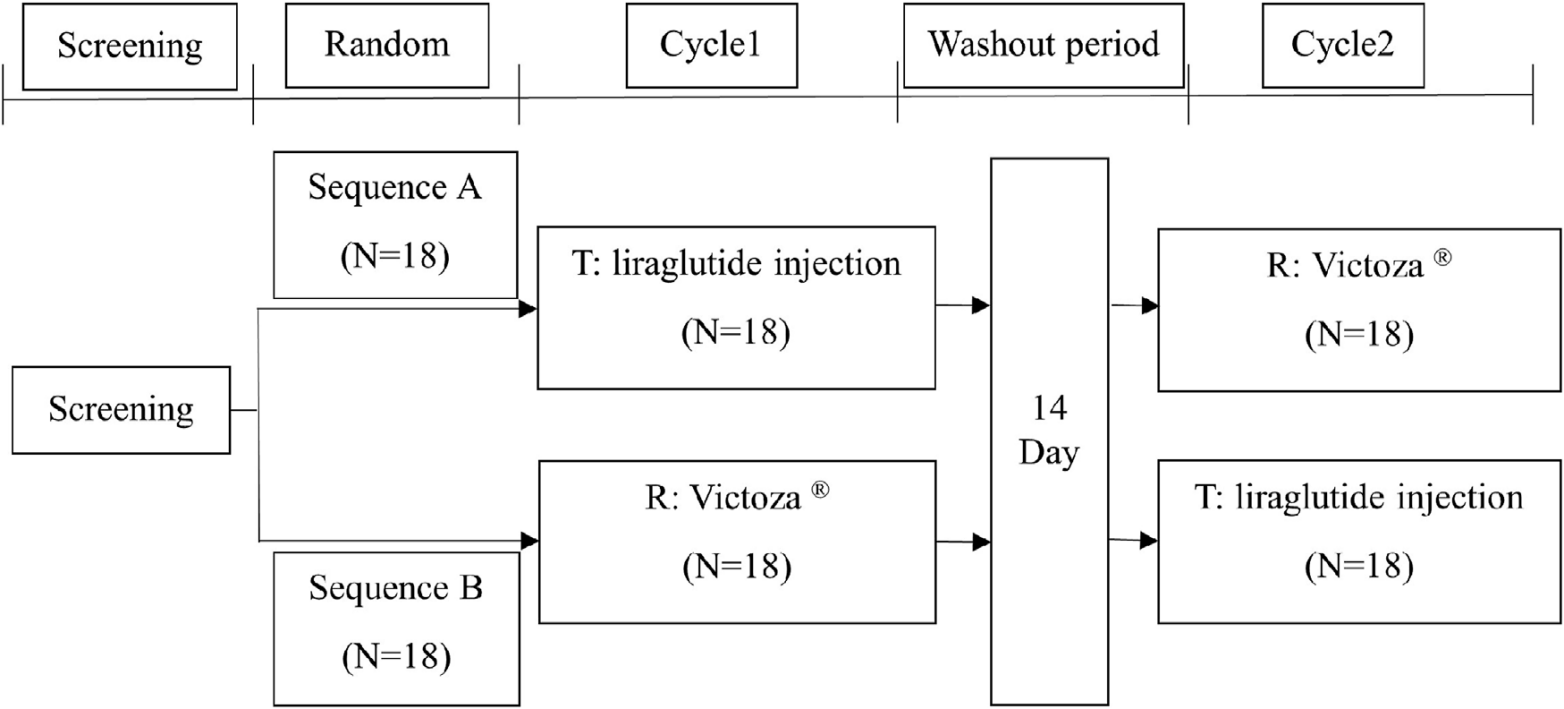
Study design and study flow diagram. T, test drug; R, reference drug.

This trial was designed as a randomized, open, two-cycle, self-crossover trial. Screening was performed within 28 days prior to dosing, and eligible subjects were admitted to the Phase I Clinical Trial Ward in the afternoon of the first day prior to dosing and fasted for 10 h overnight after dinner. A total of 36 subjects were enrolled in this trial and were randomized in a 1:1 ratio into two groups of 18 subjects each, with a single subcutaneous injection of 0.6 mg of liraglutide injection or Victoza^®^ within 10 cm around the umbilicus. A total of 18 subjects received the test formulation in the first cycle, and the other 18 subjects received the reference formulation for administration and were crossover-administered for the second cycle of the study after a 14-day washout period. Drinking water was restricted for 1 h before and 1 h after dosing, water was drunk as needed at all other times, and a standardized nutritious meal was uniformly provided at least 4 h after dosing. The subjects were discharged from the hospital on the third day after dosing. Follow-up examinations were performed 72 h after the last dose of the second cycle. The detailed study design is shown in Figure 1.

### 2.4 Estimation of the sample size

In this study, an auto-crossover design was used, with pharmacokinetic parameters (AUC and C_max_) as the main analytical indexes, assuming unilateral *α* = 0.05, *β* = 0.2, intra-variation coefficient (CV) = 11% [with reference to the AUC intra-CV coefficient of 11% of the instruction manual of Victoza^®^ (US Food and Drug Administration, 2017)], and the ratio of the mean of the test drug to the reference drug was 0.90–1.10, and the bioequivalent interval was 80.00%–125.00%. PASS (version 11.0.7) software was used to calculate the estimated sample size of 13 cases. However, considering the unknown intra-individual coefficient of variation of C_max_ and the shedding rate, the final sample size was set to 36 cases.

### 2.5 Pharmacokinetic assessment

During each cycle, 5 mL of blood was taken within 1 h prior to initiation of liraglutide injection (pre-dose) and at 1, 3, 5, 6, 7, 8, 9, 10, 11, 12, 14, 16, 24, 36, 48, 60, and 72 h after administration and placed in test tubes containing EDTA dipotassium anticoagulant. After collection, the tubes were inverted six times, centrifuged at 4°C, 1,460 g for 10 min, and the plasma was aliquoted and placed it the numbered cryopreservation tube. Plasma samples were stored in a refrigerator at −80°C before analysis.

Plasma concentrations of liraglutide were analyzed using a validated, sensitive, and specific liquid chromatography–tandem mass spectrometry (LC-MS/MS) method developed by Shanghai WuXi AppTec New Drug Development Co. (Shanghai, China). Waters ACQUITY UPLC (Waters Corporation, United States) and AB Triple Quad 6500+ Mass Spectrometer (SCIEX Technologies, United States) combined with an electrospray ionization source were used for LC-MS/MS analysis. The internal standard of liraglutide was developed in-house. The chromatographic separation was performed on a C18 column (Waters) at 60°C with a flow rate of 600 μL/min. The mobile phase A was 0.025% formic acid aqueous solution (v/v), and the mobile phase B was 0.025% formic acid acetonitrile aqueous solution (v/v). The linear range, limit of quantification, accuracy, precision, recovery, selectivity, and stability of the method were verified. The linear range of the analytical method for the determination of liraglutide in plasma was 1.00–250 ng/mL, and the lower limit of quantification (LLOQ) was 1.00 ng/mL. The CVs of the intra- and inter-run accuracies were less than 10% and 13%, respectively. Intra- and inter-run accuracies were within 100% ± 10% over the entire assay range. The analyte recoveries were determined using three gradients, high, medium, and low, with mean values of 48.0%, 47.6%, and 57.8%, respectively, corresponding to CVs of less than 8% and 57.3 for IS, corresponding to a CV of 5.1%. All six batches of matrices passed the interference with endogenous substances in the Chinese human plasma blank matrices. The short-term stability (ULOQ) of the working solution of the analytes to be tested was 146.9 h at room temperature in an aqueous mixture of formic acid and acetonitrile; the stability of the biological samples after preparation was 21.5 h at room temperature. During analysis, plasma concentrations of liraglutide below the limit of quantification were listed as zero and missing values in PK samples before and after Tmax, respectively. In the concentration data listings, all missing data are indicated as “-” or NA (not applicable).

The pharmacokinetic parameters, including T_max_, C_max_, AUC_0-t_, AUC_0-∞_, t_1/2z_, AUC__%Extrap_, V_z_/F, and CL_z_/F, were calculated using the non-compartmental model of WinNonlin 7.0. Descriptive statistical analysis was performed on PK parameters, and the arithmetic mean, standard deviation, coefficient of variation, maximum, minimum, and geometric mean of each parameter were calculated. If AUC__%Extrap_ >20%, the subject’s AUC_0-∞_, t_1/2z_, V_z_/F, and CL_z_/F were not to be used for descriptive statistical analysis.

### 2.6 Statistical analysis

Based on the blood concentration data, SAS 9.4 was used for statistical analysis. C_max_, AUC_0-t_, AUC_0-∞_, t_1/2z_, CL_z_/F, and V_z_/F were logarithmically transformed and then analyzed using variance analysis (ANOVA). In the ANOVA model, sequence, drug, and period are regarded as fixed effects, and subject sequences are regarded as random effects. Two one-sided tests were performed for each parameter, and 90% CI of the geometric mean ratio (GMR) of the pharmacokinetic parameters (T/R) was calculated. If the ratio was within the equivalence interval of 80.00%–125.00%, then the pharmacokinetics of reference liraglutide were similar to those of test liraglutide.

### 2.7 Safety and tolerability assessment

Subjects receiving the study drug were included in the safety assessment. The safety assessment included physical examination, vital signs (pre-dose and 1, 3, 12, 24, 48, and 72 h after administration of the study drug in the first and second cycles), laboratory tests, 12-lead electrocardiograms (pre-dose and 3, 24, 48, and 72 h after administration of the study drug in the first and second cycles), and blood glucose monitoring. Laboratory safety assessments were completed at the laboratory of Zhongshan Hospital, Fudan University, including routine blood, blood biochemistry, thyroid function, coagulation function, and urine routine tests. All adverse reactions were coded using MedDRA 21.0 terminology and categorized and analyzed according to SOC/PT. To detect the occurrence of hypoglycemic events, a drop of spent blood left at the tip of the syringe after collection of the pharmacokinetic sample was used for blood glucose measurement at the bedside while the subject was taking the Victoza^®^ drug in each cycle, and blood glucose values were recorded at pre-dose, 1, 3, 5, 6, 7, 8, and 9 h post-dose, respectively (the results shown in the Supplementary Material).

## 3 Results

### 3.1 Subject demographics

**FIGURE 2:**
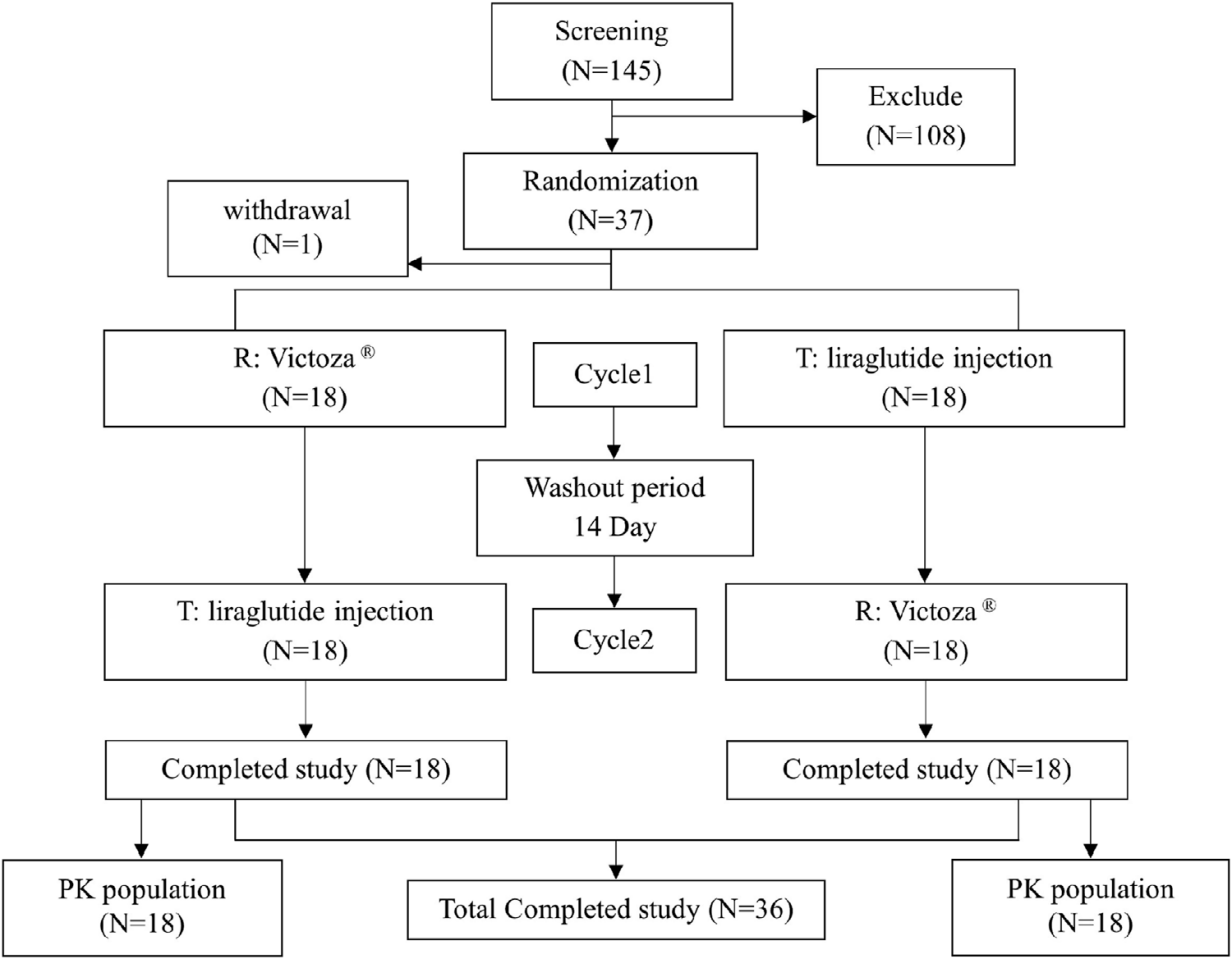
Flowchart of subject screening. PK, pharmacokinetic; T, test drug; R, reference drug.

A total of 145 subjects were screened, with 1 subject withdrawing from the trial prior to dosing after randomization, for an actual total of 36 subjects (22 male and 14 female subjects) who were randomly assigned and completed the study (Figure 2). All 36 subjects were enrolled in the safety set, the PK concentration set, the PK parameter set, and the bioequivalence analysis set. The demographics of the 36 subjects are shown in Table 1.

**TABLE 1:** Demographic characteristics of all the subjects in the study.

Parameter	T-R (N = 18)	R-T (N = 18)	Total (N = 36)
Age (years)			
N	18	18	36
Mean ± SD	28.1 ± 6.83	26.5 ± 6.17	27.3 ± 6.47
Min–Max	19–41	18–39	18–41
Gender, n (%)			
Male	11 (61.1)	11 (61.1)	22 (61.1)
Female	7 (38.9)	7 (38.9)	14 (38.9)
Ethnicity, n (%)			
Han	17 (94.4)	17 (94.4)	34 (94.4)
Other nationalities	1 (5.6)	1 (5.6)	2 (5.6)
Height (cm)			
Mean ± SD	166.19 ± 6.591	165.39 ± 7.712	165.79 ± 7.082
Min–Max	151–178.2	152.4–177.8	151–178.2
Weight (kg)			
Mean ± SD	62.86 ± 5.684	61.21 ± 7.998	62.04 ± 6.890
Min–Max	51.9–71.3	50.2–75.9	50.2–75.9
BMI (kg/m^2^)			
Mean ± SD	22.76 ± 1.666	22.31 ± 1.703	22.53 ± 1.677
Min–Max	20.3–25.2	19.4–25.0	19.4–25.2

N, number of subjects; SD, standard deviation; BMI, body mass index; T, test drug, R, reference drug.

### 3.2 Pharmacokinetic assessment

**FIGURE 3:**
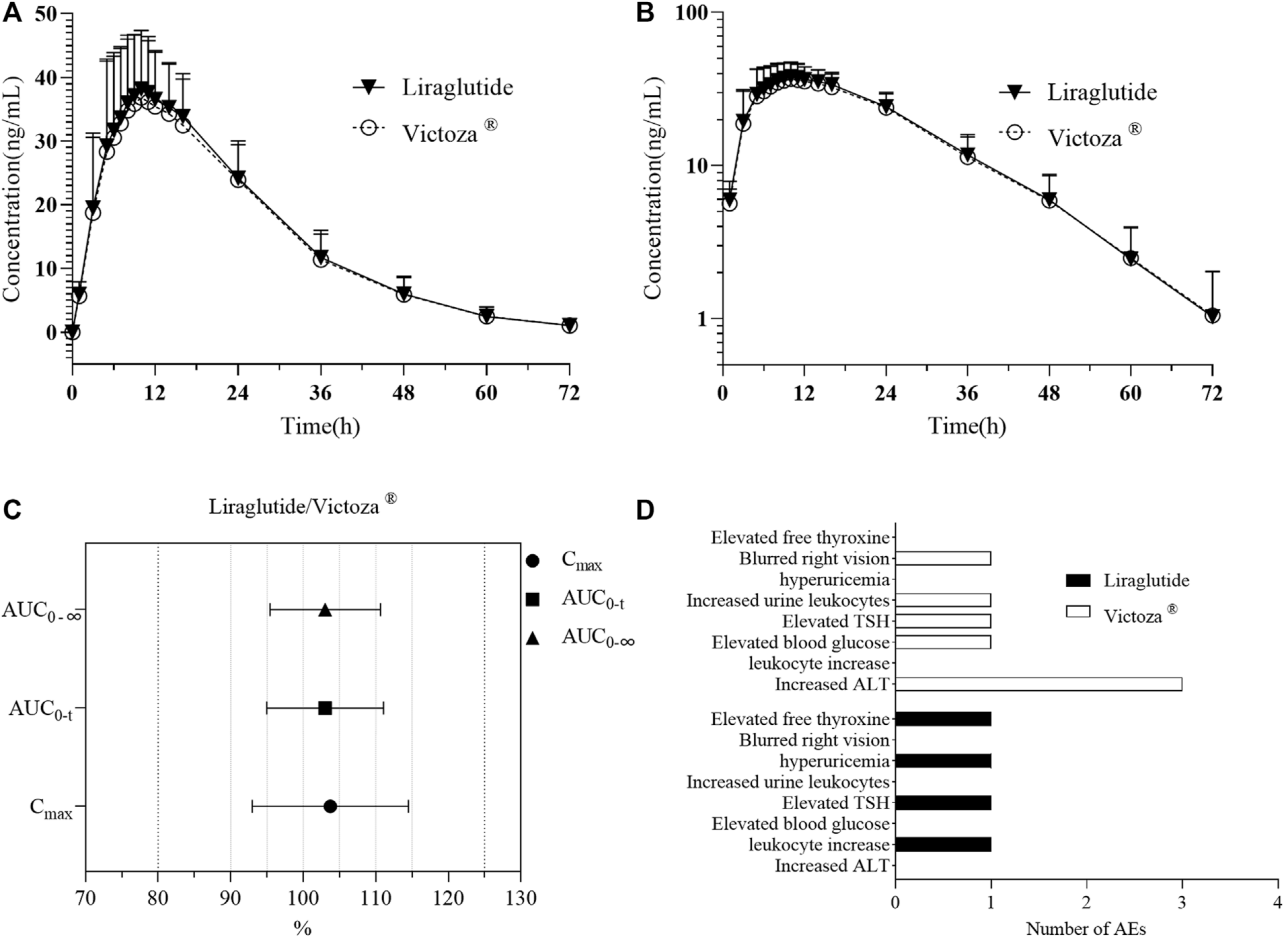
PK analysis of liraglutide and Victoza^®^. **(A)** Mean blood concentration (±SD) time profile (arithmetic mean) after subcutaneous injection of liraglutide and Victoza^®^. **(B)** Mean blood concentration (±SD) time profile (logarithmically transformed) after subcutaneous injection of liraglutide and Victoza^®^. **(C)** Bioequivalence analysis of liraglutide and Victoza^®^, which showed the ratio of major PK parameters of liraglutide and Victoza^®^ was within the preset acceptable range of 80%–125%, indicating bioequivalence of the two agents. **(D)** Number of AEs after treatment with liraglutide and Victoza^®^. AUC_0-t_, AUC of the analyte in plasma in the interval from time zero to the last measurable concentration; AUC_0-∞_, AUC of the analyte in plasma in the interval from time zero to infinity; C_max_, maximum drug concentration observed in plasma; liraglutide, the test drug; Victoza^®^, the reference drug; TSH, thyroid-stimulating hormone; ALT, alanine aminotransferase.

Pharmacokinetic parameters were calculated using the WinNonlin 7.0 non-compartmental model. Pharmacokinetic parameters C_max_, AUC_0-t_, AUC_0-∞_, T_1/2z_, T_max_, λz, AUC__%Extrap_, V_z_/F, and CL_z_/F of liraglutide in plasma of 36 subjects eligible for the PK parameter set were tabulated and analyzed with descriptive statistics. Descriptive statistics of liraglutide pharmacokinetic parameters for the PK parameter set population are shown in Table 2. The mean blood concentration–time curves and the mean blood concentration–time semi-log curves are shown in Figures 3A, B.

**TABLE 2:** Summary of treatment-emergent adverse events.

Symptom	Liraglutide (N = 36)		Victoza^®^ (N = 36)	
	Instance	Number of cases (n%)	Instance	Number of cases (n%)
Total AEs	3	8.33	7	19.44
AEs of grade 1 and above	0	0	0	0
Increased alanine aminotransferase	0	0	3	8.33
Leukocyte increase	1	2.78	0	0
Elevated blood glucose	0	0	1	2.78
Elevated hypersensitivity thyroid-stimulating hormone	1	2.78	1	2.78
Increased urine leukocytes	0	0	1	2.78
Hyperuricemia	1	2.78	0	0
Blurred right vision	0	0	1	2.78
Elevated free thyroxine	1	2.78	0	0
Serious AEs	0	0	0	0
AEs leading to exit	0	0	0	0

Liraglutide, the test drug; Victoza^®^, the reference drug; n%, the proportion of adverse reactions that occurred in all subjects treated with liraglutide and Victoza^®^.

### 3.3 Biosimilarity analysis

A multifactorial ANOVA was performed to analyze the main pharmacokinetic parameters C_max_, AUC_0-t_, and AUC_0-∞_ of liraglutide in the reference drug and the test drug. The results showed that there were no statistically significant differences in the LnC_max_, LnAUC_0-t_, and LnAUC_0-∞_ of liraglutide among sequences, weekly intervals, and formulations (Table 3).

**TABLE 3:** PK parameters of the reference and subject drug groups of liraglutide injection.

Parameter (unit)	Mean ± SD (%CV) (N = 36)	
	Liraglutide	Victoza^®^
T_max_ (h)	10.00 (5, 16)	10.00 (5, 16)
C_max_ (ng/mL)	40.05 ± 12.07 (30.1)	38.83 ± 12.76 (32.9)
AUC_0-t_ (h·ng/mL)	1085.17 ± 229.77 (21.2)	1058.05 ± 246.17 (23.3)
AUC_0-∞_(h·ng/mL)	1112.78 ± 236.32 (21.2)	1084.76 ± 252.86 (23.3)
λ_z_ (×10^−2^ 1/h)	6.89 ± 1.40 (20.4)	6.72 ± 1.47 (22.0)
T_1/2z_(h)	10.40 ± 1.79 (17.2)	10.72 ± 1.98 (18.4)
AUC__%Extrap_ (%)	2.49 ± 0.97 (39.0)	2.47 ± 0.85 (34.6)
V_z_/F (mL)	8341.87 ± 1866.85 (22.4)	8990.84 ± 2847.11 (31.7)
CL_z_/F (mL/h)	562.90 ± 120.94 (21.5)	582.49 ± 137.57 (23.6)

T_max_, time from administration to observation of maximum plasma analyte concentration, which is expressed as the median (minimum, maximum) value; C_max_, maximum drug concentration observed in plasma; AUC_0-t_, AUC of analytes in plasma during the time interval from time zero to the last measurable concentration; AUC_0-∞_, area under the curve from zero to infinity; λz, elimination of the rate constant, the slope of the terminal segment of the semi-logarithmic drug–time curve calculated using linear regression; T_1/2z_, elimination of terminal half-life; AUC__%Extrap_, ((AUC_0-∞_−AUC_0-t_)/AUC_0-∞_) × 100; V_z_/F, apparent volume of distribution; CL_z_/F, apparent clearance; SD, standard deviation; CV, coefficient of variation; N, number of subjects; liraglutide, the test drug; Victoza^®^, the reference drug.

Based on the above results, the main parameters of C_max_, AUC_0-t_, and AUC_0-∞_ were tested using the double one-sided *t*-test and confidence interval method, and the GMR of the main PK parameters (C_max_, AUC_0-t_, and AUC_0-∞_) and their 90% CIs calculated for 36 subjects after subcutaneous injection of liraglutide injection are shown in Table 4. The 90% CIs for the GMR of C_max_, AUC_0-t_, and AUC_0-∞_ for liraglutide all fall exactly between 80.00% and 125.00%, suggesting that the pharmacokinetics of the subject drug and the reference drug are similar (Figure 3C).

**TABLE 4:** Results of multifactorial ANOVA for liraglutide.

Variable	*p*-value		
	LnC_max_	LnAUC_0-t_	LnAUC_0-∞_
Dosing sequence	0.562	0.514	0.524
Dosing cycles	0.385	0.943	0.937
Formulation factors	0.142	0.116	0.095

LnC_max_, log-transformed Cmax; LnAUC_0-t_, log-transformed AUC_0-t_; LnAUC_0-∞_, log-transformed AUC_0-∞_.

### 3.4 Adverse events (AEs)

In this trial, the vital signs of 36 subjects randomly enrolled in the group fluctuated within the normal physiological range, no clinically significant abnormalities were found, and no abnormalities were found around the umbilicus after administration. A total of 11 cases of adverse reactions occurred (Table 5; Figure 3D), except for blurred right vision. All AEs may be related to medication, all of which occurred after administration. Three subjects (8.33%) in the test preparation group had four adverse reactions, of which two (5.56%) subjects had possible related adverse reactions three times; seven subjects in the reference preparation group had adverse reactions seven times, and four cases (11.11%) of the subjects had possible related adverse reactions four times. No serious adverse reactions occurred in the two groups, and no adverse reactions leading to withdrawal from the trial occurred. All the AEs were mild, and subjects recovered without any treatment.

**TABLE 5:** Results of bioequivalence evaluation of liraglutide.

Parameter (unit)	Geometric mean and ratio (N = 36)			CV (%)	90% confidence interval (%)	Power of test (%)
	Liraglutide	Victoza^®^	(T/R) %			
C_max_ (ng/mL)	38.61	37.22	103.73	10.38	99.54–108.10	>99.99
AUC_0-t_ (h·ng/mL)	1062.05	1031.05	103.01	7.81	99.85–106.26	>99.99
AUC_0-∞_ (h·ng/mL)	1089.18	1057.15	103.03	7.39	100.04–106.11	>99.99

C_max_, maximum drug concentration observed in plasma; AUC_0-t_, AUC of analytes in plasma during the time interval from time zero to the last measurable concentration; AUC_0-∞_, area under the curve from zero to infinity; N, number of subjects; SD, standard deviation; BMI, body mass index; T, test drug; R, reference drug; CV, coefficient of variation.

## 4 Discussion

Liraglutide, a GLP-1 analog with 97% amino acid homology to endogenous substances (Knudsen and Lau, 2019), has been used as a GLP-1 receptor agonist for the treatment of type 2 diabetes (T2D) for more than 10 years (Knudsen and Lau, 2019; Nauck et al., 2021a), and its safety, tolerability, and pharmacodynamics have been effectively demonstrated clinically. Studies have shown that liraglutide contributes to the comprehensive regulation of glucose metabolism in patients with T2D, attenuates the symptomatic risk of diabetic complications, which mainly includes a beneficial effect on the cardiometabolic system through the improvement of endothelial function, vascular inflammation, vasodilatory effects, diuretic effects, and optimization of lipid parameters (Campbell and Drucker, 2013; Herzlinger and Horton, 2013), and may result in a low probability of the development or worsening of renal disorders (Marso et al., 2016), contributing to weight loss in overweight or obese T2D patients (Davies et al., 2015; Garvey et al., 2020), as well as relieving symptoms in cases of NAFLD (Armstrong et al., 2016; Petit et al., 2017). It is worth adding that the trial drug from Huadong Pharmaceutical Company in this study was approved for marketing in March 2023 for use in adults with T2D, and the marketing authorization for this formulation regarding the indication of obesity or overweight was approved by the NMPA in July 2023. This study is a randomized, open, two-cycle, crossover trial design to investigate the bioequivalence and safety of the test drug and the original drug.

The starting dose of liraglutide was 0.6 mg per day, according to the specification of Victoza^®^ (Novo Nordisk, 2011). The lower dose on the steeper portion of the exposure–effect curve in healthy subjects was chosen to assess the difference between the subject drug and the reference drug (CDER, 2014), and therefore, 0.6 mg was selected as the dose to be administered in this comparative trial. The washout period between the two drugs was 14 days, much longer than the 7-day half-life of liraglutide, which is in line with the dosing requirements (Jacobsen et al., 2016; Knudsen and Lau, 2019). Analysis of PK parameters in the male and female populations showed that factors related to age, gender, race, and body weight had no clinically significant effect on pharmacokinetics (Jacobsen et al., 2016). A total of 37 healthy subjects were screened for successful enrollment in the trial, of which one withdrew prior to the first cycle of dosing, which had no impact on the trial sample size requirements. A total of 36 subjects were randomly enrolled in this trial, and the data obtained were suitable for safety analysis, PK parameter analysis, and bioequivalence analysis.

Previous studies (US Food and Drug Administration, 2017) have shown that when a single dose of 0.6 mg was administered subcutaneously, the maximum concentration was reached 8–12 h after dosing, the mean peak (Cmax) and total exposure (AUC) of liraglutide were 35 ng/mL and 960 h ng/mL, respectively, the mean apparent volume of distribution was approximately 13 L, and the mean apparent clearance of a single dose of liraglutide following subcutaneous administration was approximately 1.2 L/h, which was in general agreement with the results of previous phase I studies of liraglutide in healthy Chinese subjects (Jiang et al., 2011) and in the present study. With respect to the BE study, the 90% CI for the GMR of AUC_0-t_, C_max_, and AUC_0-∞_ was between 80% and 125%, which is considered bioequivalent (Mai et al., 2020). In the present study, the GMR of C_max_, AUC_0-t_, and AUC_0-∞_ after subcutaneous injection of 0.6 mg liraglutide injection (3 mL: 18 mg) in the 36 healthy subjects was 103.73%, 103.01%, and 103.03%, respectively, with 90% CIs ranging from 99.54% to 108.10%, 99.85% to 106.26%, and 100.04% to 106.11%, all of which fell exactly between 80.00% and 125.00%, while the PK parameter curves and log-transformed curves of the two drug concentrations were basically the same. This indicates that the pharmacokinetics of the test drug and the reference drug are similar after subcutaneous injection of 0.6 mg liraglutide injection (3 mL: 18 mg) in healthy subjects.

In five double-blind clinical trials of 26 weeks or longer, the incidence of gastrointestinal adverse reactions in patients was reported to be 41% in the liraglutide treatment group and was dose-related (US Food and Drug Administration, 2017). This is consistent with the pharmacodynamic profile of GLP-1R agonist-related drugs. Clinical data show that gastrointestinal side effects are the most common and frequent adverse reactions following the use of GLP-1R agonist analogs, manifesting as nausea, vomiting, diarrhea, and possibly cholelithiasis, which generally appear to be dose-related and transient, disappearing with continuation of therapy (Fineman et al., 2004). There have also been a few reports of acute pancreatitis (Li et al., 2014), retinopathy (Ipp et al., 2017), and immunogenic reactions following drug administration (Aroda and Ratner, 2011). A total of eight AEs were observed in this study (see Table 2), with no gastrointestinal reactions detected, mostly abnormal laboratory tests, which may be related to factors such as the fact that healthy subjects themselves were more tolerant compared to patients and the fact that it was a single, small-dose administration with a limited number of subjects. One adverse event of elevated blood glucose occurred in the reference formulation in a subject whose baseline blood glucose level was close to the upper limit of the blood glucose reference concentration, which may be related to the fluctuation in his blood glucose level. Elevated thyroid hormone levels were observed in both the test drug and the reference drug, which is consistent with the results of adverse events in earlier clinical trials of liraglutide (US Food and Drug Administration, 2017). Overall, four AEs occurred in three subjects in the test drug group, and seven AEs occurred in seven subjects in the reference drug group, with no serious AEs/reactions in either group and no reactions to AEs leading to withdrawal from the trial, indicating that the test drug and the reference drug were well tolerated in terms of safety.

Immunogenicity, assessed primarily by detecting the incidence of ADA and Nab, is a unique and important research component of biomolecule drugs. In five clinical trials of 26 weeks or longer (US Food and Drug Administration, 2017), approximately 50%–70% of Victoza-treated patients were tested for anti-liraglutide antibodies at the end of treatment. Approximately 8.6% of the liraglutide-treated group were detected with low-titer (no serum concentration dilution required) anti-liraglutide antibodies. When comparing all antibody-positive and all antibody-negative patients, antibody formation was not accompanied by a reduction in the efficacy of liraglutide on mean HbA1c, and there was no reduction in HbA1c in the patients with the highest titers of anti-liraglutide antibodies detected. This suggests that antibody formation does not lead to a reduction in the efficacy of liraglutide. In our bioequivalence study, the test drug and Victoza^®^ injection belong to the same small-molecule biological peptide with similar drug metabolism characteristics and immunogenicity. Therefore, this study did not design a test for immunogenicity.

## 5 Conclusion

This study compared the bioequivalence of two liraglutide injections with the test drugs developed by Hangzhou Zhongmei Huadong Pharmaceutical Co. Ltd. and Hangzhou Jiuyuan Gene Engineering Co. Ltd. and the original drug produced by Novo Nordisk, Denmark. The results of the clinical trial showed that the pharmacokinetics of the two formulations was bioequivalent and both showed a favorable safety profile. These data will facilitate the clinical application of the test drug as a biosimilar.

## Data Availability

The raw data supporting the conclusion of this article will be made available by the authors without undue reservation.
