# Supplementary material for: Evaluating the bioequivalence and safety of liraglutide injection versus Victoza® in healthy Chinese subjects: a randomized, open, two-cycle, self-crossover phase I clinical trial
Source: Front Pharmacol. 2023 Dec 22;14:1326865. doi: 10.3389/fphar.2023.1326865 (PMC10766854; doi:10.3389/fphar.2023.1326865)
Supplement: Supplementary file 1 [file Table1.DOCX]

**Subject exclusion criteria:**

Subjects will be excluded from the study if they meet any of the following criteria at the screening visit or baseline visit (Day -1):

1. History of clinically significant drug allergy or atopic allergic disease (asthma, urticaria, eczematous dermatitis) or known allergy to the test medication or similar medication.
2. Current or recent 6-month history of dyspepsia, esophageal reflux, gastric hemorrhage, or peptic ulcer disease, frequent heartburn on more than one occasion (weekly), or any surgical procedure (e.g., cholecystectomy) that may interfere with absorption of medications.
3. A history or family history (grandparents, parents, and siblings) of medullary thyroid cancer (MTC), or a genetic disorder predisposing to induction of MTC.
4. Signs of dermatitis or skin abnormalities in and around the site of administration.
5. Participation in any clinical trial within 3 months prior to the trial.
6. History of major surgery within 3 months prior to the trial or scheduled to undergo surgery within 14 days of administration.
7. History of blood loss or donation within 90 days (counted from 1 day prior to dosing) with a loss or donation of more than 300mL of blood.
8. Strenuous exercise within 48 h prior to administration, or other factors affecting drug absorption, distribution, metabolism and excretion.
9. Use of medication within 2 weeks prior to study drug administration or receipt of any prescription medication within one month prior to Day 1 of the study. The exception is aspirin or acetaminophen, which are allowed within 1 day prior to screening and prior to the baseline evaluation (Day -1).
10. Smoke more than 5 cigarettes or equivalent amount of tobacco per day or cannot quit smoking during the trial.
11. Daily intake of caffeinated beverages exceeding 6 units per day (1 unit = 120 mg of caffeine).
12. A history of regular alcohol consumption with more than 7 drinks per week for women or more than 14 drinks per week for men (1 drink = 5 oz (150mL) of wine = 12 oz (360mL) of beer = 1.5 oz (45mL) of spirits) within the 28-day Screening Visit or a positive breath test for alcohol at the Screening Visit and the Baseline Visit.
13. History of psychotropic substance abuse or positive urine screening test for substance abuse or drugs at screening visit/baseline visit.
14. Positive human immunodeficiency virus antibody (HIV antibody), serum syphilis spirochete antibody (Anti-TP), hepatitis B virus surface antigen (HBsAg), or hepatitis C virus antibody (anti-HCV).
15. Women with serum HCG ≥ 5 mIU/ml at screening visit or baseline visit and who are breastfeeding.
16. Sitting systolic blood pressure >140 mmHg or <80 mmHg, diastolic blood pressure >90 mmHg or <50 mmHg, pulse <50 beats/minute or >100 beats/minute; or systolic blood pressure in the range of 80-90 mmHg and diastolic blood pressure in the range of 50-60 mmHg, accompanied by symptoms of hypotension, such as dizziness, black eye, limb weakness, and so on.
17. Subjects with partners who refuse to use effective contraception (including barrier IUDs combined with spermicides, oral contraceptives, and barrier measures) between screening and 3 months after study completion.
18. Those who, in the opinion of the investigator, are not suitable for participation in this trial.
