# Supplementary figures and images for: Evaluating the bioequivalence and safety of liraglutide injection versus Victoza® in healthy Chinese subjects: a randomized, open, two-cycle, self-crossover phase I clinical trial
Source: Front Pharmacol. 2023 Dec 22;14:1326865. doi: 10.3389/fphar.2023.1326865 (PMC10766854; doi:10.3389/fphar.2023.1326865)

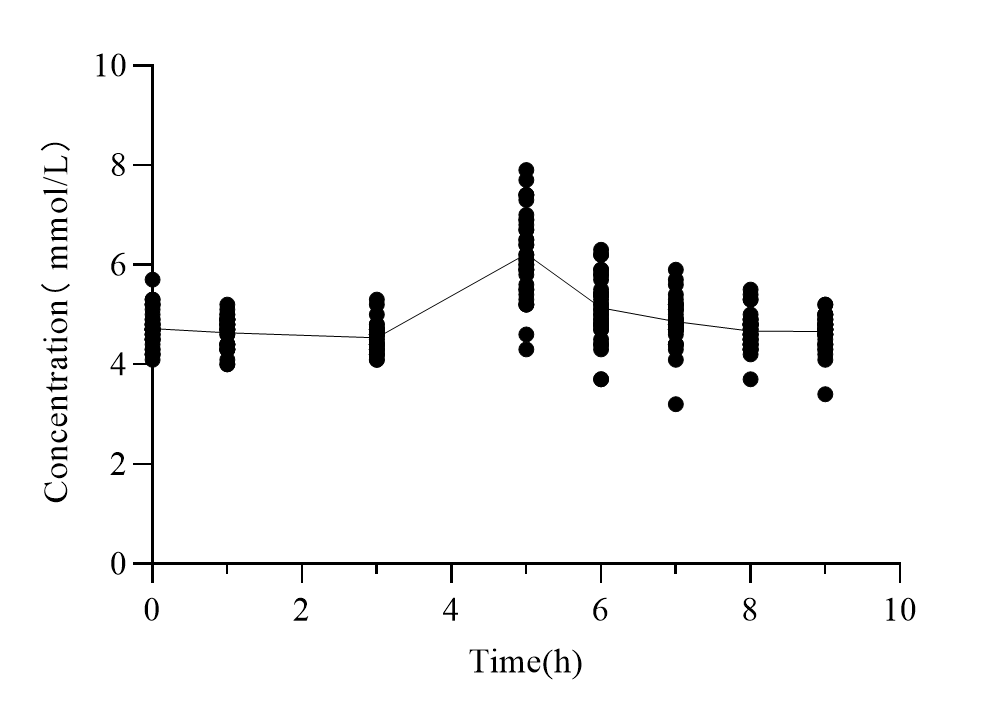

Supplement: Supplementary file 2 [file Image1.TIF]
